# Bone Marrow Stromal Cells With Exercise and Thyroid Hormone Effect on Post-Stroke Injuries in Middle-aged Mice

**DOI:** 10.32598/bcn.9.10.355

**Published:** 2019-01-01

**Authors:** Kobra Akhoundzadeh, Abedin Vakili, Hamid Reza Sameni

**Affiliations:** 1. Physiology Research Center, Semnan University of Medical Sciences, Semnan, Iran.; 2. Department of Nursing, School of Nursing & Midwifery, Qom University of Medical Sciences, Qom, Iran.; 3. Nervous System Stems Cells Research Center, Department of Anatomical Sciences, School of Medicine, Semnan University of Medical Sciences, Semnan, Iran

**Keywords:** Cerebral ischemia, Combination, Bone marrow stromal cells, Thyroid hormone, Exercise, Apoptosis, Glial fibrillary acidic protein, Middle-aged, Mice

## Abstract

**Introduction:**

Based on our previous findings, the treatment of stem cells alone or in combination with thyroid hormone (T_3_) and mild exercise could effectively reduce the risk of stroke damage in young mice. However, it is unclear whether this treatment is effective in aged or middle-aged mice. Therefore, this study designed to assess whether combination of Bone Marrow Stromal Cells (BMSCs) with T_3_ and mild treadmill exercise can decrease stroke complications in middle-aged mice.

**Methods:**

Under laser Doppler flowmetry monitoring, transient focal cerebral ischemia was produced by right Middle Cerebral Artery Occlusion (MCAO) for 45 min followed by 7 days of reperfusion in middle-aged mice. BMSCs (1×10^5^) were injected into the right cerebral ventricle 24 h after MCAO, followed by daily injection of triiodothyronine (T_3_) (20 μg/100 g/d SC) and 6 days of running on a treadmill. Infarct size, neurological function, apoptotic cells and expression levels of Glial Fibrillary Acidic Protein (GFAP) were evaluated 1 week after stroke.

**Results:**

Post-ischemic treatment with BMSCs or with T_3_ and or mild treadmill exercise alone or in combination did not significantly change neurological function, infarct size, and apoptotic cells 7 days after ischemia in middle-aged mice (P>0.05). However, the expression of GFAP significantly reduced after treatment with BMSCs and or T_3_ (P<0.01).

**Conclusion:**

Our findings indicate that post-stroke treatment BMSCs with exercise and thyroid hormone cannot reverse neuronal damage 7 days after ischemia in middle-aged mice. These findings further support that age is an important variable in stroke treatment

## Highlights

- Our previous research indicates that treatment of stem cells alone or in combination with thyroid hormone, T_3_, and exercise can reduce brain damage in young mice.
- It is unclear whether this treatment protocol can be effective in middle-aged mice.
- Post-stroke treatment bone marrow stem cells with exercise and T_3_ cannot reverse neuronal damage in middle-aged mice.
- The therapeutic response to bone marrow stem cells, T_3_ and exercise is different between young and middle-aged mice.

## Plain Language Summary

Cerebrovascular (CV) diseases are the second cause of mortality in Iran and developed countries. Despite the great advances in medicine in recent decades, no definitive treatment for the CV diseases have not been found yet. Neuro-scientists seek to find a new therapeutic strategy for cerebral stroke such as cell therapy. Our previous study results reveal that combination therapy (stem cells, and thyroid hormone, and exercise) effectively reduces stroke damage and recovers neurological disorders in young mice. Considering that major stroke cases occur in older humans people, this question arises whether this treatment protocol can also be effective in middle-aged mice. In this study, cerebral stroke was created by blocking the middle cerebral artery in middle-aged mice that is almost equivalent to a 60 years old human. A combination therapy (stem cells, and thyroid hormone and exercise) was begun 24 hours after the stroke and continued daily for 6 consecutive days. Infarct size, sensory and motor function disorders and molecular cellular changes were measured 7 days after the stroke. Our results indicate that, unlike younger animals, this treatment protocol does not have a therapeutic effect in middle-aged mice.

## 1. Introduction

Ischemic stroke occurs more often in older people, (Darsalia, Heldmann, Lindvall, & Kokaia, 2005) and is an important cause of mortality and morbidity in them (Eady et al., 2014). According to clinical and animal studies, outcome and mortality after brain damage and the efficacy of neuroprotective agents are age dependent (Tang et al., 2014; Zhu et al., 2005). It has been proven that aging worsens brain injury and diminishes functional recovery of post-ischemic damage in rodent models of cerebral stroke (Badan et al., 2003; Hiona & Leeuwenburgh, 2004; Li, Zheng, & Carmichael, 2005; Popa-Wagner et al., 2007; Zhuo et al., 2010). Moreover, it has also been shown that the risk of stroke increases with aging in humans and recovery after stroke lessens in older patients compared with younger ones (Bejot et al., 2010; Marini et al., 2001).

Older age is associated with inadequate collateral circulation, and consequently adverse tissue outcome and unfavorable clinical outcome after stroke (Arsava et al., 2013). Although, stroke is known as an age-related disorder, most of the experimental stroke-related studies have been done in young or adult animals. This is probably one of the reasons why stroke therapeutic interventions, effective in experimental animals, fail in clinical situations (Tang et al., 2014). Therefore, use of older animal models for stroke research is clinically more applicable (Balseanu, Buga, & Popa-Wagner, 2010).

Thyroid hormone is necessary for maturation of the brain in the fetal period and cerebral function in adulthood through controlling the expression of many genes (Diez et al., 2008). According to recent animal and clinical studies, thyroid hormone may be beneficial in the management of cerebral stroke (Alevizaki, Synetou, Xynos, Pappa, & Vemmos, 2007; Bunevicius, Iervasi, & Bunevicius, 2015; O’Keefe et al., 2015). In addition, thyroid hormone may participate in regulation proliferation, migration, and maturation of neural stem cells in the brain (Abbaszadeh, Tiraihi, Delshad, Zadeh, & Taheri, 2013; Ambrogini et al., 2005; Benvenuti et al., 2008). Moreover, exercise can reduce the complication of cerebral stroke in humans and animals through various mechanisms, including boosting survival of neurons, promoting production of new neurons and blood vessels, enhancing synaptic plasticity, and inhibition of neuronal apoptosis (Gao et al., 2014; Ma, Qiang, & He, 2013; Seo et al., 2014).

Stem cell therapy is a new therapeutic strategy to improve neurogenesis and repair brain ischemic injuries that may be potentiated when combined with other neuroprotective agents (Jin et al., 2010; Popa-Wagner, Filfan, Uzoni, Pourgolafshan, & Buga, 2015). In this regard, some animal investigations have reported that stem cell therapy alone or in combination with other neuro-protective interventions such as exercise, can effectively attenuate ischemic injury through various mechanisms in young animal model of stroke (Bang, Lee, Lee, & Lee, 2005; Hicks et al., 2007; Jin et al., 2010; Lee et al., 2013; Zhang et al., 2015).

Our previous findings showed that post-ischemic treatment of BMSCs combined with exercise, and thyroid hormone more efficiently reduced brain damage in young mice (Akhoundzadeh et al., 2017). However, it is unclear whether this treatment approach can be effective in aged or middle-aged mice. Moreover, there is little and sometimes contradictory data regarding the effects of stem cells therapy alone or in combination with other neuroprotective agents on recuperation of cerebral ischemic damage in middle-aged and aged animal (Jin et al., 2010; Tang et al., 2014; Tatarishvili et al., 2014; Wagner, Bojko, & Peters, 2012). Therefore, this study designed to assess whether combination of BMSCs with T_3_ and mild treadmill exercise can attenuate stroke-induced injury 7 days after ischemia in middle-aged mice.

## 2. Methods

### 2.1. Animals and ethics

Middle-aged male Swiss albino mice (11–12 mon, 35–40 g) were provided from animal center of Semnan University of Medical Sciences (SUMS), Semnan, Iran. The mice were housed in a standard condition and food and water were available ad libitum. All tests were done in conforming to the Research Ethics Committee (ethical code number: 93.475925) and national policy for approaching animal research.

### 2.2. Focal cerebral ischemia

Mice were anesthetized by ketamine (60 mg/kg IP) and xylazine (10 mg/kg IP) and transient focal cerebral ischemia was induced using the intraluminal filament method (Panahpour, Dehghani, & Bohlooli, 2014; Vakili, Nekooeian, & Dehghani, 2004). Under the surgical microscope, an incision was made on the midline of neck and then right common carotid artery and its branches were isolated. Using Laser Doppler blood Flow (LDF) (Moor Instruments DRT4, England), a silicone-coated 8-0 monofilament was applied to create focal cerebral ischemia. Right middle cerebral artery was blocked for 45 min and recirculation was done for 7 days. Body temperature was kept at 37±0.5°C throughout the experiment by an electrical blanket.

### 2.3. Cell extraction and transplantation

Bone marrow was harvested aseptically from tibia and femur bones of mice by pushing bone marrow out with Dulbecco’s modified Eagle’s medium (DMEM, low glucose), supplemented with Fetal Bovine Serum (FBS) and penicillin/streptomycin (100 U/mL and 100 g/mL, respectively), to a tissue culture flask. The flask was incubated at 37°C (5% CO_2_).

After 72–96 h, the cells were washed twice with PBS to remove non-attached cells. After reaching 80%–90% confluence, the plastic-adherent Bone Marrow Stromal Cells (BMSCs) were isolated with 0.25% trypsin-EDTA and then replaced for 3–5 passages (Chen et al., 2001; Ikeda et al., 2005; Sung et al., 2008). The viability of BMSCs, detected with trypan blue exclusion method, was more than 90%. Under aseptic conditions, 2 μL of cell suspension (1×10^5^ BMSCs) or PBS (as vehicle) was injected into the right lateral ventricle (0.9 mm right, 0.1 mm posterior, and 3.1 mm deep relative to the bregma) 24 h after right Middle Cerebral Artery Occlusion (MCAO) (Paxinos & Franklin, 2001).

### 2.4. Exercise training and thyroid hormone injection protocols

Treadmill exercise was initiated 24 h after ischemia for 30 min and continued daily for 6 consecutive days (Zhang et al., 2016). Mild exercise included running at 3 m/min for 5 min, 5 m/min for 5 min, and then 8 m/min for 20 min at 0° slope (18). Thyroid hormone (T_3_, Sigma, USA) was administered at 20 μg/100g/d (Simonides et al., 2008) subcutaneously for six consecutive days, initiating 24 h following ischemia.

### 2.5. Neurobehavioral test

Neurological evaluation was done 7 days after ischemia in all experimental groups as presented in Table 1 (Li et al., 2000; Rehni, Singh, Jaggi, & Singh, 2007). Neurological function was rated between 0 and 14 (normal score=0; maximum deficit score=14). A score of 10 to 14 is severe; 5 to 9 moderate; and 1to 4 mild. A researcher, who was blind to the experimental groups, evaluated neurological disorder. Beam balance test assesses sensorimotor function and balance in rodents. In this test, animals must walk across an elevated balance beam (length: 100 cm, width: 1.2 cm, height: 50 cm). Scoring was done in all of experimental groups as presented in Table 2 (Carter, 1999; Shen et al., 2006; Southwell, Ko, & Patterson, 2009).

**Table 1.** Neurobehavioral test

**Neurobehavioral Items**		**Behavioral Test**	**Score**
Motor tests	Raising the mouse by the tail	Flexion of forelimb	1
		Flexion of hindlimb	1
		Head moved >10 ° to vertical axis within 30 s	1
		Inability to walk straight	1
	Placing the mouse on the floor	Circling toward the paretic side	1
		Falling down to the paretic side	1
		Immobility and staring	1
	Abnormal movements	Tremor (wet-dog-shakes)	1
		Myodystony, irritability, seizures, myoclonus	1
Sensory tests Visual and tactile placing (limb placing test to detect visual and superficial sensory)	Moving the mouse laterally toward the table	Reaching the table slowly with limbs or cannot place at all	1
	Proprioceptive test (deep sensory), pushing the paw against the table edge to stimulate limb muscles	Losing the resistance Reflexes	1
		Absence of pinna reflex (a head shake when touching the auditory meatus)	1
		Absence of corneal reflex (an eye blink when lightly touching the cornea with cotton)	1
		Absence of startle reflex (a motor response to a brief loud noise from snapping a clipboard paper)	1
		Maximum points	14

Beam balance test

**Table 2.** Beam balance test

**Beam Balance Test**	**Points**
Balances with steady posture	0
Grasps side of beam	1
Hugs the beam and one limb falls down from the beam	2
Hugs the beam and two limbs fall down from the beam (>60 s)	3
Attempts to balance on the beam but falls off (>40 s)	4
Attempts to balance on the beam but falls off (>20 s)	5
Falls off: no attempt to balance or hang on to the beam (<20 s)	6
Maximum points	6

### 2.6. Experimental groups

Infarct size, neurological disorder, apoptotic cells and GFAP-positive cells were evaluated at day 7 after ischemia in eight different groups as followed (n=4–6): Group 1 (sham-operated group), surgery without MCAO; Group 2 (control group), stroke-subjected animals received PBS (2 μL) intracerebroventricular (ICV) at 24 h after MCAO; Group 3 (BMSCs group), stroke-subjected animals received BMSCs (105 cells, 2 μL ICV) at 24 h after MCAO; Group 4 (T_3_ group), stroke-subjected animals received T_3_ (20 μg/100g/d, SC) daily for 6 days, starting 24 h after ischemia; Group 5 (EX group), stroke-subjected animal was forced to do mild treadmill exercise (running at 3 m/min for 5 min, 5 m/ min for 5 min, and then 8 m/min for 20 min at 0° slope) that was started at 24 h after ischemia and continued up to the seventh day after MCAO; Group 6 (BMSCs+T_3_ group), stroke-subjected animals received both BMSCs and T_3_; Group 7 (BMSCs+EX group), stroke-subjected animals received both BMSCs and exercise; Group 8 (BMSCs+T_3_+EX group), animals received all BMSCs, T_3_ and exercise.

### 2.7. Brain damage measurements and TUNEL assay

Under deep anesthesia, saline and then 4% paraformaldehyde were perfused transcardially at 7 days after MCAO in all experimental groups. After decapitation, the brains were removed, immersed in 4% paraformaldehyde and then embedded in paraffin wax. Nine coronal sections of each animal brain (10-μm thick) were provided, starting 100-μm interval from bregma −1 to +1 of the ischemic hemisphere by a microtome for brain damage measurement, TUNEL and immunohistochemistry assay.

Three coronal brain sections (10-μm thick) were used to measure the infarct area with cresyl fast violet staining (Nissl staining). Briefly, according to the protocol, after deparaffinized and hydration in xylene, ethanol and distilled water, brain slices were immersed in 0.5% cresyl fast violet (Sigma, St. Louis, MO) at 65°C for 7 min followed by being immersed in acetic acid 0.25% in ethanol 50% for 1 to 2 seconds and then washed. Afterward, sections were photographed using a digital camera (Cannon, Japan) and infarcted areas were calculated by an image analysis system (Motic Images Plus 2.0) and data were reported as the percentage of the infracted area.

Three coronal brain sections (10-μm thick) were used for TUNEL apoptotic cell detection using an in Situ Cell Death Detection kit, POD (Roche Diagnostic GmbH, Germany) (Dubska, Matalova, & Misek, 2002). According to the protocol, after deparaffinization, the tissue was incubated with proteinase K, permeabilized with permeabilization solution, and incubated in TUNEL solution. After each stage, washing was performed with PBS. The number of TUNEL-positive cells (brown) was visualized using a Reichert microscope (USA) with a 40× magnification and counted by a blinded investigator on six non-overlapping visual fields for each section.

### 2.8. Immunohistochemistry assay

Three equal coronal sections of each sample were used for immunohistochemistry assay. Endogenous peroxidase activity of brain sections was extinguished with 3% H2O2 in PBS (60 min) and non-specific binding was blocked with 10% goat serum, 0.5% Triton X-100, and 0.1% bovine serum albumin in PBS (30 min). The slices were incubated with primary antibody (rabbit anti-GFAP, 1:100, Biorbyt, UK) overnight at 4°C followed by incubation with secondary antibody (biotinylated goat anti-rabbit immunoglobulin IgG,1:100, Biorbyt, UK) for 2 h at room temperature. 3, 3’-Diaminobenzidine (DAB, Sigma, Germany) was used for staining GFAP-positive cells and counterstaining was done with hematoxylin eosin. The number of GFAP-stained cells (brown) was visualized using a Reichert microscope (USA) with a 40× magnification and counted by a blinded researcher on six non-overlapping visual fields for each section.

### 2.9. Statistical analysis

The statistical tests of 1-way ANOVA were used for comparison among groups regarding infarct area, GFAP-positive and TUNEL-positive cells. The Kruskal-Wallis ANOVA on rank and Dunn’s method as post-hoc tests were used to analyze neurological scores. Results of neurological scores and beam balance are presented as median±IQR (interquartile range) and other variables as Mean±SEM. Differences were considered statistically significant at P<0.05 (SigmaStat 2.0; Jandel Scientific, Erkrath, Germany).

## 3. Results

### 3.1. Effect of combination BMSCs with T_3_ and exercise on brain lesion and neurological disorder

**Figure 1.**
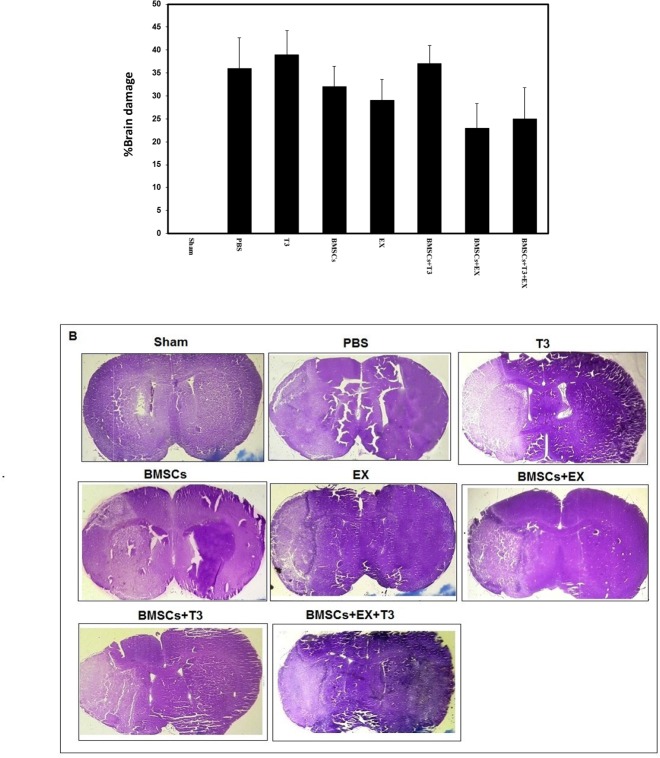
Infarct size in different groups Presence of brain damage (A) and microphotographs of cresyl violet staining in sham operated, PBS (control), BMSCs, EX, T_3_, BMSCs+T_3_, BMSCs+EX and BMSCs+EX+T_3_ groups, seven days after MCAO in mice. Values are presented as Mean±SEM.

The percentage of infarct area in the PBS control group was 36%±7 at day 7 after cerebral ischemia. Treatment with BMSCs (32%±4%) or with T_3_ (39%±5%) and or mild treadmill exercise (29%±5%) alone did not significantly change percentage of infarct size (P>0.05, Figure 1 A, B). Additionally, combination treatment of BMSCs and T_3_ (37%±4%), BMSCs and EX (23%±6%), and BMSCs and EX and T_3_ (25%±7%) did not change percentage of infarct size significantly compared with the PBS as the control group (P>0.05, Figure 1 A, B).

**Figure 2.**
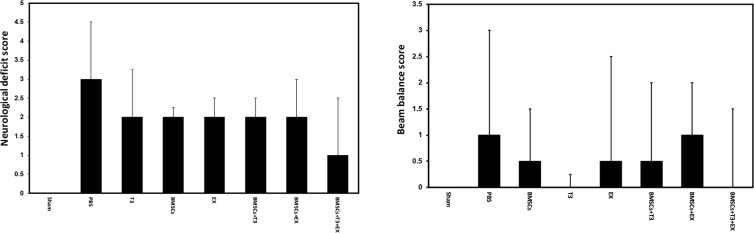
Neurological function in different groups Neurological deficit scores (A) and beam balance scores (B) in sham operated, PBS (control), BMSCs, EX, T_3_, BMSCs+T_3_, BMSCs+EX and BMSCs+EX+T_3_ groups, seven days after MCAO in mice. Values are presented as median ± IQR (interquartile range).

Neurological dysfunction score was 3±1.5 in the PBS group at day 7 after cerebral ischemia. Treatment with BMSCs (2±0.25) or with T_3_ (2±1.25) and or mild tread-mill exercise (2±0.5) alone did not improve neurological outcome (H=6.233 with 6 degrees, P=0.398, Figure 2 A). Furthermore, combination treatment of BMSCs and T_3_ (2±0.5), BMSCs and EX (2±1), and BMSCs and EX and T_3_ (1±1.5) did not significantly improve neurological deficits compared with the PBS as the control group (H=6.233 with 6 degrees, P=0.398, Figure 2 A). In addition, scores of beam balance test were not different between experimental groups (H=3.932 with 6 degrees, P=0.686, Figure 2 B).

### 3.2. Effect of BMSCs combination with T_3_ and exercise on apoptotic cells

**Figure 3.**
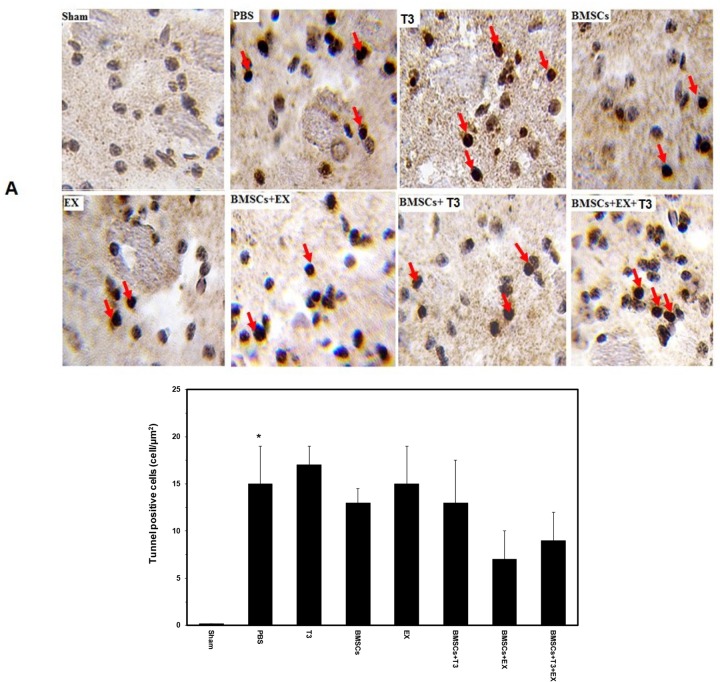
Apoptotic cells in different groups TUNEL staining image (A) and quantitative analysis of number of TUNEL-positive cells (B) in the sham-operated, PBS (control), BMSCs, EX, T_3_, BMSCs+T_3_, BMSCs+EX and BMSCs+EX+T_3_ groups, seven days after MCAO in mice. The number of TUNEL-positive cells (brown) was visualized using a Reichert microscope with a 40× magnification. Values are presented as Mean±SEM.

The number of TUNEL-positive cells (apoptotic cells) in the PBS control group was 15±4 at day 7 after cerebral ischemia. Treatment with BMSCs (13± 2) or with T_3_ (17±2) and or mild treadmill exercise (18±4) alone did not change content of TUNEL-positive cells (P>0.05, Figure 3 A, B). Additionally, combination treatment of BMSCs and T_3_ (13±5), BMSCs and EX (7±3), and BMSCs and EX and T_3_ (9±3) did not change the number of TUNEL-positive cells significantly compared with the PBS as the control group (P>0.05, Figure 3 A, B).

### 3.3. Effect of BMSCs combination with T_3_ and exercise on GFAP expression

**Figure 4.**
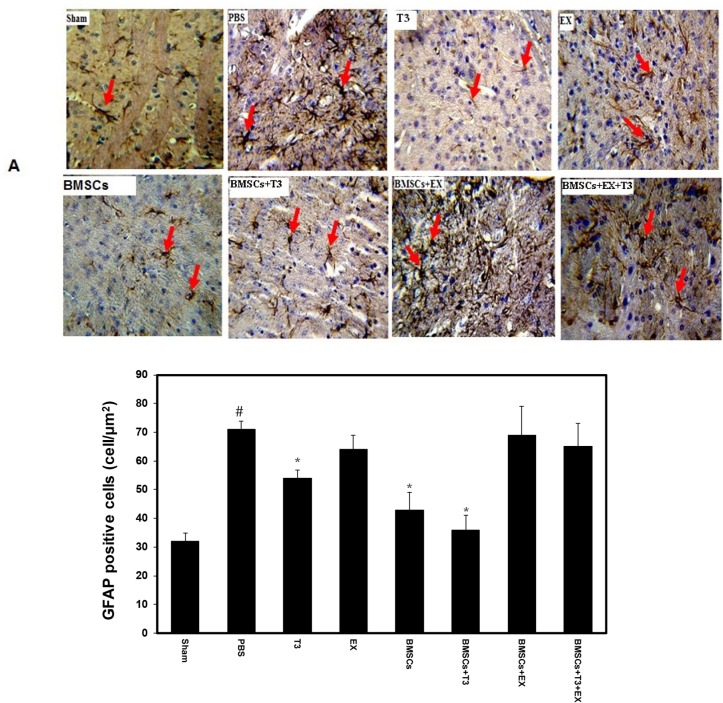
GFAP positive cells in different groups Photomicrographs of GFAP-positive cells (A) and quantitative analysis of the number of GFAP-positive cells (B) in the sham-operated, PBS (control), BMSCs, EX, T_3_, BMSCs+T_3_, BMSCs+EX and BMSCs+EX+T_3_ groups, seven days after MCAO in mice. Values are presented as Mean±SEM. *P<0.001 compared to the PBS group. # P<0.001, compared to the sham-operated group. The number of GFAP-stained cells (brown) was visualized using a Reichert microscope with 40× magnification.

Cerebral ischemia considerably enhanced the GFAP staining in the PBS group as control (90±10) compared with the sham group (42±1) (P<0.001, Figure 4 A, B). Treatment with BMSCs (43±6) and or T_3_ (54±2.9) significantly reduced GFAP-stained cells (P<0.001, Figure 4 A, B). Additionally, combination treatment of BMSCs+T_3_ (36±5) significantly declined GFAP-stained cells compared with the PBS (P<0.001, Figure 4 A, B).

## 4. Discussion

The main findings of this research suggest that BMSCs transplantation alone or in combination with T_3_ or mild treadmill exercise did not significantly change infarct size, neurological function and apoptosis seven days after stroke in middle-aged mice. Moreover, other finding of the present study indicate that post-ischemic treatment only with T_3_, BMSCs and BMSCs+T_3_ significantly reduced GFAP as a marker of astroglial activation and gliosis.

Extensive neuroprotective drugs and or molecules were discovered in pre-clinical stroke studies in young animals, but most of them failed to produce a fruitful results in clinical studies. One possible reason for this discrepancy may relate to the fact that most experimental stroke studies have been conducted on young animals, while stroke often occurs in aged people. However, use of aged animals in stroke research is associated with troubles such as higher mortality and morbidity, which makes researchers reluctant to study on aged animals. Nevertheless, using results of studies on aged animals is more applicable to clinical situation of stroke. Therefore, in the present study, we used mice aged 11 to 12 months, which corresponds to humans aged approximately 60 years.

Our data indicated that treatment with BMSCs or mild exercise failed to decrease the brain injury and recovery of neurological function one week after stroke in middle-aged mice. This result is in agreement with previous investigations, that showed bone marrow mononuclear cell transplantation or exercise failed to recover the brain damage in aged rats in experimental stroke (Leasure & Grider, 2010; Wagner et al., 2012). Moreover, our data were supported by previous research studies indicating that with increasing age, vulnerability of the brain to ischemia increases and response to treatment decreases (Arsava et al., 2013; Balseanu et al., 2010; Popa-Wagner, Buga, Tica, & Albu, 2014; Tan, Li, & Kelly, 2009). Also, our finding is in line with a study indicating that treatment with apocynin (an inhibitor of NADPH oxidase), unlike with young mice, worsens brain damage and increases the mortality rate in aged mice (Kelly et al., 2009). However, results of the present study is in contrast with our recent findings in young mice (Akhoundzadeh et al., 2017).

Apoptosis is an important cellular mechanism that may be involved in the development of secondary ischemic damage after stroke (Popa-Wagner et al., 2007). Our study indicated that the number of apoptotic cells considerably increased one week after ischemia in middle-aged mice. However, treatment with BMSCs alone or combined with thyroid hormone and or exercise could not reduce the apoptosis in ischemic area one week after stroke in the present study that was in line with the amount of brain damage and neurological function.

Our findings showed that treatment alone with BMSCs and or T_3_ and combination therapy BMSCs+T_3_ significantly attenuated stroke-induced astrogliosis 7 days after stroke in middle-aged mice. Similar to our findings, a number of studies reported that stem cell transplantation (Leu et al., 2010; Song, Jue, Cho, & Kim, 2015) and or thyroid hormone therapy could decrease GFAP expression and astrogliosis after stroke (Leu et al., 2010; Song et al., 2015). Although in the present study, GFAP expression decreased in BMSCs and or T_3_ groups, this decline was not associated with improved brain damage or neurological function. It is probable that other amplified pathologic mechanisms following stroke in aged brain impede therapeutic effects of decrease in astrogliosis (Wagner et al., 2012).

Taken together, comparison between the present results with our previous findings in young mice (Akhoundzadeh et al., 2010) indicates that therapeutic response to combination therapy with BMSCs, T_3_ and exercise is different between young and middle-aged groups. Therefore, we re-emphasize that in studies of experimental models of stroke, findings obtained from aged animals are more relevant to clinical situation.

In the present study, high mortality was also a problem and about 68% of animals died before the seventh day and were excluded from the study that was the limitation of this study. We suggest that studies with larger sample size be done in aged and middle-aged animals.

Findings of the present study demonstrated that post-stroke treatment BMSCs with T_3_ or mild exercise have no beneficial effects in the recovery of ischemic injury seven days after MCAO in middle-aged mice. We suggest that in stroke research, aged animals be used to evaluate the effect of anti-ischemic potential agents against brain injury, as these results are more clinically applicable.

## Ethical Considerations

### Compliance with ethical guidelines

All tests were done in conforming to the Research Ethics Committee (Ethical code No.: 93.475925) and national policy for approaching animal research.

## References

[B1] Abbaszadeh H. A. Tiraihi T. Delshad A. R. Zadeh M. S. Taheri T. (2013). Bone marrow stromal cell transdifferentiation into oligodendrocyte-like cells using triiodothyronine as a inducer with expression of platelet-derived growth factor α as a maturity marker. Iranian Biomedical Journal, 17(2), 62–7. [PMID] [PMCID] 23567847 10.6091/ibj.11162.2013 PMC3677678

[B2] Akhoundzadeh K. Vakili A. Sameni H. R. Vafaei A. A. Rashidy-Pour A. Safari M. (2017). Effects of the combined treatment of bone marrow stromal cells with mild exercise and thyroid hormone on brain damage and apoptosis in a mouse focal cerebral ischemia model. Metabolic Brain Disease, 32(4), 1267–77. [DOI:10.1007/s11011-017-0034-0] 28547077

[B3] Alevizaki M. Synetou M. Xynos M. Pappa T. Vemmos K. N. (2007). Low triiodothyronine: A strong predictor of outcome in acute stroke patients. European Journal of Clinical Investigation, 37(2), 651–7. [DOI:10.1111/j.1365-2362.2007.01839.x] [PMID] 17635576

[B4] Ambrogini P. Cuppini R. Ferri P. Mancini C. Ciaroni S. Voci A. (2005). Thyroid hormones affect neurogenesis in the dentate gyrus of adult rat. Neuroendocrinology, 81(4), 244–53. [DOI:10.1159/000087648] [PMID] 16113586

[B5] Arsava E. Vural A. Akpinar E. Gocmen R. Akcalar S. Oguz K. (2013). The detrimental effect of aging on leptomeningeal collaterals in ischemic stroke. Journal of Stroke & Cerebrovascular Disease, 23(3), 421–6. 10.1016/j.jstrokecerebrovasdis.2013.03.014 23583014

[B6] Badan I. Buchhold B. Hamm A. Gratz M. Walker L. C. Platt D. (2003). Accelerated glial reactivity to stroke in aged rats correlates with reduced functional recovery. Journal of Cerebral Blood Flow & Metabolism, 23(3), 845–54. [DOI:10.1097/01.WCB.0000071883.63724.A7] [PMID] 12843788

[B7] Balseanu A. Buga A. Popa-Wagner A. (2010). Cellular response to cerebral ischemia during aging. Current Science, 35(4), 209–18.

[B8] Bang O. Y. Lee J. S. Lee P. H. Lee G. (2005). Autologous mesenchymal stem cell transplantation in stroke patients. Annals of Neurology, 57(6), 874–82. [DOI:10.1002/ana.20501] [PMID] 15929052

[B9] Bejot Y. Rouaud O. Jacquin A. Osseby G. V. Durier J. Manckoundia P. (2010). Stroke in the very old: Incidence, risk factors, clinical features, outcomes and access to resources—A 22-year population-based study. Cerebrovascular Disease, 29(2), 111–21. [DOI:10.1159/000262306] [PMID] 19955734

[B10] Benvenuti S. Luciani P. Cellai I. Deledda C. Baglioni S. Saccardi R. (2008). Thyroid hormones promote cell differentiation and up-regulate the expression of the seladin-1 gene in in vitro models of human neuronal precursors. Journal of Endocrinology, 197(2), 437–46. [DOI:10.1677/JOE-07-0324] [PMID] 18434374

[B11] Bunevicius A. Iervasi G. Bunevicius R. (2015). Neuroprotective actions of thyroid hormones and low-T3 syndrome as a biomarker in acute cerebrovascular disorders. Expert Reviewi of Neurotherapy, 15(3), 315–26. [DOI:10.1586/14737175.2015.1013465] [PMID] 25673072

[B12] Carter R. (1999). Characterization of progressive motor deficits in mice transgenic for the human Huntington’s disease mutation. Journal of Neuroscience, 19(4), 3248–57. [DOI:10.1523/JNEUROSCI.19-08-03248.1999] [PMID] 10191337 PMC6782264

[B13] Chen J. Li Y. Wang L. Lu M. Zhang X. Chopp M. (2001). Therapeutic benefit of intracerebral transplantation of bone marrow stromal cells after cerebral ischemia in rats. Journal of the Neurological Sciences, 189(1), 49–57. [DOI:10.1016/S0022-510X(01)00557-3] 11535233

[B14] Darsalia V. Heldmann U. Lindvall O. Kokaia Z. (2005). Stroke-induced neurogenesis in aged brain. Stroke, 36(1), 1790–5. [DOI:10.1161/01.STR.0000173151.36031.be] [PMID] 16002766

[B15] Diez D. Grijota-Martinez C. Agretti P. De Marco G. Tonacchera M. Pinchera A. (2008). Thyroid hormone action in the adult brain: gene expression profiling of the effects of single and multiple doses of triiodo-L-thyronine in the rat striatum. Endocrinology, 149(8), 3989–4000. [DOI:10.1210/en.2008-0350] [PMID] 18467437

[B16] Dubska L. E. Matalova I. Misek I. (2002). Detection of apoptosis in paraffin embedded tissues: the influence of tissue type and fixation. Acta Veterina, 71(1), 529–33. [DOI:10.2754/avb200271040529]

[B17] Eady T. N. Khoutorova L. Obenaus A. Mohd-Yusof A. Bazan N. G. Belayev L. (2014). Docosahexaenoic acid complexed to albumin provides neuroprotection after experimental stroke in aged rats. Neurobiology Disesea, 62(1), 1–17. [DOI:10.1016/j.nbd.2013.09.008] [PMID] [PMCID] PMC3877728 24063996

[B18] Gao Y. Zhao Y. Pan J. Yang L. Huang T. Feng X. (2014). Treadmill exercise promotes angiogenesis in the ischemic penumbra of rat brains through caveolin-1/VEGF signaling pathways. Brain Research, 1585, 83–90. [DOI:10.1016/j.brainnres.2014.08.032] 25148708

[B19] Hicks A. U. Hewlett K. Windle V. Chernenko G. Ploughman M. Jolkkonen J. (2007). Enriched environment enhances transplanted subventricular zone stem cell migration and functional recovery after stroke. Neuroscience, 146(1), 31–40. [DOI:10.1016/j.neuroscience.2007.01.020] 17320299

[B20] Hiona A. Leeuwenburgh C. (2004). Effects of age and caloric restriction on brain neuronal cell death/survival. Annual New York Academy of Science, 1019, 96–105. [DOI:10.1196/annnals.1297.018] [PMID] 15247000

[B21] Ikeda N. Nonoguchi N. Zhao M. Watanabe T. Kajimoto Y. Furutama D. (2005). Bone marrow stromal cells that enhanced fibroblast growth factor-2 secretion by herpes simplex virus vector improve neurological outcome after transient focal cerebral ischemia in rats. Stroke, 36(4), 2725–30. [DOI:10.1161/01.STR.0000190006.88896.d3] [PMID] 16282547

[B22] Jin K. Mao X. Xie L. Greenberg R. B. Peng B. Moore A. (2010). Delayed transplantation of human neural precursor cells improves outcome from focal cerebral ischemia in aged rats. Aging Cell, 9(6), 1076–83. [DOI:10.1111/j.1474-9726.2010.00638.x] [PMID] [PMCID] 20883527 PMC2980591

[B23] Kelly K. Li X. Tan Z. VanGilder R. Rosen C. Huber J. (2009). NOX2 inhibition with apocynin worsens stroke outcome in aged rats. Brain Research, 6(1292), 165–72. [DOI:10.1016/j.brainres.2009.07.052] [PMID] [PMCID] PMC2751637 19635468

[B24] Leasure J. L. Grider M. (2010). The effect of mild post-stroke exercise on reactive neurogenesis and recovery of somatosensation in aged rats. Experimental Neurology, 226(13), 58–67. [DOI:10.1016/j.expneurol.2010.08.003] 20696163

[B25] Lee D. H. Lee J. Y. Oh B. M. Phi J. H. Kim S. K. Bang M. S. (2013). Functional recovery after injury of motor cortex in rats: Effects of rehabilitation and stem cell transplantation in a traumatic brain injury model of cortical resection. Childs Nerve System, 29(4), 403–11. [DOI:10.1007/s00381-012-1969-4] 23180314

[B26] Leu S. Lin Y. C. Yuen C. M. Yen C. H. Kao Y. H. Sun C. K. (2010). Adipose-derived mesenchymal stem cells markedly attenuate brain infarct size and improve neurological function in rats. Journal of Translational Medicine, 8(63), 1–16. [DOI:10.1186/1479-5876-8-63] 20584315 PMC2913939

[B27] Li S. Zheng J. Carmichael S. T. (2005). Increased oxidative protein and DNA damage but decreased stress response in the aged brain following experimental stroke. Neurobiology of Disease, 18(3), 432–40. [DOI:10.1016/j.nbd.2004.12.014] [PMID] 15755669

[B28] Li Y. Chopp M. Chen J. Wang L. Gautam S. C. Xu Y. X. (2000). Intrastriatal transplantation of bone marrow nonhematopoietic cells improves functional recovery after stroke in adult mice. Journal of Cerebral Blood Flow & Metabolism, 20(9), 1311–9. [DOI:10.1097/00004647-200009000-00006] [PMID] 10994853

[B29] Ma Y. Qiang L. He M. (2013). Exercise therapy augments the ischemia-induced proangiogenic state and results in sustained improvement after stroke. International Journal of Molecular Sciences, 14(4), 8570–84. [DOI:10.3390/ijms14048570] 23598418 PMC3645762

[B30] Marini C. Triggiani L. Cimini N. Ciancarelli I. De Santis F. Russo T. Baldassarre M. (2001). Proportion of older people in the community as a predictor of increasing stroke incidence. Neuroepidemiology, 20(2), 91–5. [DOI:10.1159/000054766] [PMID] 11359075

[B31] O’Keefe L. M. Conway S. E. Czap A. Malchoff C. D. Benashski S. Fortunato G. (2015). Thyroid hormones and functional outcomes after ischemic stroke. Thyroid Research, 8(9), 2–5. 26157487 10.1186/s13044-015-0021-7 PMC4495802

[B32] Panahpour H. Dehghani G. A. Bohlooli S. (2014). Enal-april attenuates ischaemic brain oedema and protects the blood–brain barrier in rats via an anti-oxidant action. Clinical and Experimental Pharmacology and Physiology, 41(3), 220–6. [DOI:10.1111/1440-1681.12210] [PMID] 24471927

[B33] Paxinos G. Franklin K. B. J. (2001). The mouse brain in stereotaxic coordinates. 2nd Ed. Amsterdam: Elsevier.

[B34] Popa-Wagner A. Badan I. Walker L. Groppa S. Patrana N. Kessler C. (2007). Accelerated infarct development, cytogenesis and apoptosis following transient cerebral ischemia in aged rats. Acta Neuropathology, 113(3), 277–93. [DOI:10.1007/s00401-006-0164-7] [PMID] 17131130

[B35] Popa-Wagner A. Buga A. M. Tica A. A. Albu C. V. (2014). Perfusion deficits, inflammation and aging precipitate depressive behaviour. Biogerontology, 15(5), 439–48. [DOI:10.1007/s10522-014-9516-1] [PMID] 25033986

[B36] Popa-Wagner A. Filfan M. Uzoni A. Pourgolafshan P. Buga A. M. (2015). Poststroke cell therapy of the aged brain. Hindawi Publishing Corporation: Neural Plasticity, 2015, 7–31. [DOI:10.1155/2015/839638] [PMID] [PMCID] PMC4548142 26347826

[B37] Rehni A. K. Singh N. Jaggi A. S. Singh M. (2007). Amniotic fluid derived stem cells ameliorate focal cerebral ischaemia-reperfusion injury induced behavioural deficits in mice. Behavioural Brain Research, 183, 95–100. [DOI:10.1016/j.bbr.2007.05.028] 17619060

[B38] Seo T. B. Kim T. W. Shin M. S. Ji E. S. Cho H. S. Lee J. M. (2014). Aerobic exercise alleviates ischemia-induced memory impairment by enhancing cell proliferation and suppressing neuronal apoptosis in hippocampus. International Neurourology Journal, 18(3), 187–97. [DOI:10.5213/inj.2014.18.4.187] [PMID] [PMCID] 25562035 PMC4280438

[B39] Shen L. H. Li Y. Chen J. Zhang J. Vanguri P. Borneman J. (2006). Intracarotid transplantation of bone marrow stromal cells increases axon-myelin remodeling after stroke. Neuroscience, 137(2), 393–9. [DOI:10.1016/j.neuroscicence.2005.08.092] [PMID] 16298076

[B40] Simonides W. S. Mulcahey M. A. Redout E. M. Muller A. Zuidwijk M. J. Visser T. J. (2008). Hypoxia-inducible factor induces local thyroid hormone inactivation during hypoxic-ischemic disease in rats. The Journal of Clinical Investigation, 118(3), 975–83. [DOI:10.1172/JCI32824] 18259611 PMC2230657

[B41] Song M. Jue S. S. Cho Y. A. Kim E. C. (2015). Comparison of the effects of human dental pulp stem cells and human bone marrow-derived mesenchymal stem cells on ischemic human astrocytes in vitro. Journal of Neuroscience Research, 93(4), 973–83. [DOI:10.1002/jnr.23569] [PMID] 25663284

[B42] Southwell A. Ko J. Patterson P. (2009). Intrabody gene therapy ameliorates motor, cognitive, and neuropathological symptoms in multiple mouse models of Huntington’s disease. Neuroscience, 29(6), 13589–602. [DOI:10.1523/JNEURORSCI.4286-09.2009] [PMID] [PMCID] 19864571 PMC2822643

[B43] Sung J. H. Yang H. M. Park J. B. Choi G. S. Joh J. W. Kwon C. H. (2008). Isolation and characterization of mouse mesenchymal stem cells. Transplantation Proceedings, 40(4), 2649–54. [DOI:10.1016/j.transproceed.2008.08.009] [PMID] 18929828

[B44] Tan Z. Li X. Kelly K. (2009). Plasminogen activator inhibitor type 1 derived peptide, EEIIMD, diminishes cortical infarct but fails to improve neurological function in aged rats following middle cerebral artery occlusion. Brain Research, 24(1281), 84–90. [DOI:10.1016/j.brainres.2009.05.042] [PMID] [PMCID] PMC2848998 19465008

[B45] Tang Y. Wang J. Lin X. Wang L. Shao B. Jin K. (2014). Neural stem cell protects aged rat brain from ischemia–reperfusion injury through neurogenesis and angiogenesis. Journal of Cerebral Blood Flow & Metabolism, 34(1), 1138–47. [DOI:10.1038/jcbfm.2014.61] [PMID] [PMCID] 24714034 PMC4083376

[B46] Tatarishvili J. Oki K. Monni E. Koch P. Memanishvili T. Buga A. M. (2014). Human induced pluripotent stem cells improve recovery in stroke-injured aged rats. Restorative Neurology and Neuroscience, 32(4), 547–58. [PMID] 24916776 10.3233/RNN-140404

[B47] Vakili A. Nekooeian A. Dehghani G. A. (2004). L-NAME and 7-nitroindazole reduces brain injuries in transient focal cerebral ischemia in rat. Iranian Journal of Medical Sciences, 29(2), 109–15.

[B48] Wagner D. C. Bojko M. Peters M. (2012). Impact of age on the efficacy of bone marrow mononuclear cell transplantation in experimental stroke. Experimental & Translational Stroke Medicine, 4(17), 1–8. [DOI:10.1186/2040-7378-4-17] [PMID] 22920434 PMC3527344

[B49] Zhang Y. Cao R. Jia X. Li Q. Qiao L. Yan G. Yang J. (2016). Treadmill exercise promotes neuroprotection against cerebral ischemia-reperfusion injury via downregulation of pro-inflammatory mediators. Neuropsychiatric Disesease Treatment, 12(3), 3161–73. [DOI:10.2147/NDT.S121779] [PMID] [PMCID] PMC5161395 28003752

[B50] Zhang Y. X. Yuan M. Z. Cheng L. Lin L. Z. Du H. W. Chen R. H. (2015). Treadmill exercise enhances therapeutic potency of transplanted bone mesenchymal stem cells in cerebral ischemic rats via anti-apoptotic effects. BMC Neuroscience, 16, 56–61. [PMID] [PMCID] 26342636 10.1186/s12868-015-0196-9 PMC4560892

[B51] Zhu C. Wang X. Xu F. Bahr B. Shibata M. Uchiyama Y. (2005). The influence of age on apoptotic and other mechanisms of cell death after cerebral hypoxia–ischemia. Cell Death and Differentiation, 12(1), 162–76. [DOI:10.1038/sj.cdd.4401545] [PMID] 15592434

[B52] Zhuo Y. Li S. H. Chen M. S. Wu J. Kinkaid H. Y. M. Fazel S. (2010). Aging impairs the angiogenic response to ischemic injury and the activity of implanted cells: Combined consequences for cell therapy in older recipients. The Journal of Thoracic and Cardiovascular Surgery, 139(5), 1286–94. [DOI:10.1016/j.jtcvs.2009.08.052] [PMID] 19931095

